# DNA Entry into and Exit out of the Cohesin Ring by an Interlocking Gate Mechanism

**DOI:** 10.1016/j.cell.2015.11.030

**Published:** 2015-12-17

**Authors:** Yasuto Murayama, Frank Uhlmann

**Affiliations:** 1 The Francis Crick Institute, Lincoln’s Inn Fields Laboratory, London WC2A 3LY, UK

## Abstract

Structural maintenance of chromosome (SMC) complexes are proteinaceous rings that embrace DNA to enable vital chromosomal functions. The ring is formed by two SMC subunits, closed at a pair of ATPase heads, whose interaction is reinforced by a kleisin subunit. Using biochemical analysis of fission-yeast cohesin, we find that a similar series of events facilitates both topological entrapment and release of DNA. DNA-sensing lysines trigger ATP hydrolysis to open the SMC head interface, whereas the Wapl subunit disengages kleisin, but only after ATP rebinds. This suggests an interlocking gate mechanism for DNA transport both into and out of the cohesin ring. The entry direction is facilitated by a cohesin loader that appears to fold cohesin to expose the DNA sensor. Our results provide a model for dynamic DNA binding by all members of the SMC family and explain how lysine acetylation of cohesin establishes enduring sister chromatid cohesion.

## Highlights

• Biochemical reconstitution of DNA entry into and exit out of the cohesin ring
• DNA-sensing lysines on the Psm3/Smc3 subunit’s ATPase trigger main ring opening
• The Wapl subunit disengages an interlocking kleisin subunit “outer gate”
• A unified model for DNA entry and exit with implications for all SMC complexes

## Graphical Abstract

**Figure:**
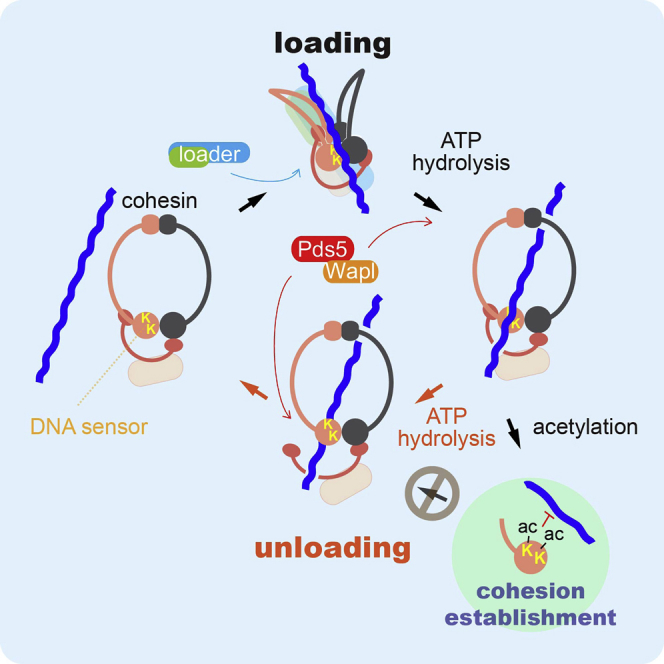

## Teaser

The biochemical reconstitution of the topological entrapment and release of DNA by the fission-yeast cohesin complex indicates that DNA sensing by lysine residues triggers entry into and exit from nested gates comprised of the cohesin complex proteins in a manner that can be regulated by acetylation.

## Introduction

Members of the structural maintenance of chromosomes (SMC) family are found in all organisms, from bacteria to human, where they play fundamental roles in chromosome organization and segregation (Hirano, 2012, Jeppsson et al., 2014, Nasmyth, 2011, Peters et al., 2008, Thadani et al., 2012). The archetypal SMC complex, like those found today in prokaryotes, probably acted akin to modern day eukaryotic condensins. They compact chromosomes and aid the resolution of replicated chromatids to facilitate their segregation. How condensin achieves this at the molecular level is not yet clear. The available evidence is consistent with a model in which condensin acts by providing dynamic interactions between pairs of its binding sites (Cheng et al., 2015, Haeusler et al., 2008).

The cohesin complex is the crucial mediator of sister chromatid cohesion and appears to be an adaptation of condensin. During the G1 phase of the cell cycle, cohesin acts similarly to condensin, being associated with chromosomes in a dynamic fashion and engaging in contacts between its binding sites (Hadjur et al., 2009, Nativio et al., 2009). During S phase, when chromosomes are replicated, a subset of cohesin is converted into a close-to-permanently chromosome-bound state by acetylation (Rolef Ben-Shahar et al., 2008, Gerlich et al., 2006, Lopez-Serra et al., 2013, Ünal et al., 2008). How cohesins that engage in interactions between newly replicated sister chromatids are singled out for acetylation remains an important question. Irrespective of the answer, stabilization of cohesin by acetylation is essential to establish enduring sister chromatid cohesion. Thus, cohesin can be thought of as a “lockable” condensin. Other contacts that cohesin makes within a chromatid, rather than between sister chromatids, are thought to contribute to gene regulation. In addition, condensin and cohesin facilitate DNA repair processes, to which also a third eukaryotic SMC complex, the Smc5-6 complex, contributes.

SMC complexes bind to DNA by topological embrace. This has first been demonstrated in the case of the budding yeast cohesin ring and has meanwhile been confirmed for condensin, the Smc5-6 complex, and a prokaryotic SMC complex (Cuylen et al., 2011, Haering et al., 2008, Kanno et al., 2015, Murayama and Uhlmann, 2014, Wilhelm et al., 2015). However, our molecular knowledge of how DNA enters and exits the SMC ring remains scarce. Understanding these DNA transitions at a molecular level will be crucial to comprehend how SMC complexes engage in chromosomal interactions and how those might be regulated.

The cohesin ring circumference is made up of a heterodimer of SMC subunits, long flexible coiled-coil proteins. The ring is closed on one side by a stable dimerization interface, known as the SMC hinge. On the other side, a pair of ABC ATPase head domains afford ATP-dependent dimerization. A kleisin subunit (Rad21 in fission yeast) bridges the ATPase heads and stabilizes their interaction. Additional subunits, Scc3 (Psc3 in fission yeast), Pds5, and Wapl make contact with the kleisin to assemble around the ATPase heads. Cohesin loading onto DNA requires ATP hydrolysis by the SMC heads and is promoted by a separate cohesin loader complex (Arumugam et al., 2003, Ciosk et al., 2000, Murayama and Uhlmann, 2014, Weitzer et al., 2003). Dynamic unloading of cohesin from chromosomes in turn is promoted by cohesin’s Wapl subunit by an as yet unknown mechanism (Bernard et al., 2008, Kueng et al., 2006, Lopez-Serra et al., 2013).

Protein engineering has previously been used to close individual cohesin ring interfaces in vivo. This led to the proposal that DNA enters the cohesin ring by opening of the SMC hinge, then exits through the SMC heads and a gap between the kleisin N terminus and Smc3 (Psm3 in fission yeast) (Buheitel and Stemmann, 2013, Gruber et al., 2006). This approach has the drawback that protein function might have been affected in ways additional to the intended interface closures. Here, to study DNA entry and exit into and out of the cohesin ring, we have taken a biochemical approach that relies as much as possible on wild-type proteins. We build on our recent biochemical reconstitution of topological cohesin loading onto DNA in vitro, which used a fission-yeast cohesin tetramer complex (Psm1, Psm3, Rad21, and Psc3) and its cohesin loader Mis4-Ssl3. We now add the two remaining cohesin subunits Pds5 and Wapl (Bernard et al., 2008, Tanaka et al., 2001, Tomonaga et al., 2000) to investigate the complete DNA entry and exit cycle. This reveals striking similarities between both directions of DNA transport and leads us to propose a unified model for DNA entry into and exit out of the cohesin ring.

## Results

### Wapl Promotes DNA Entry into the Cohesin Ring

**Figure 1:**
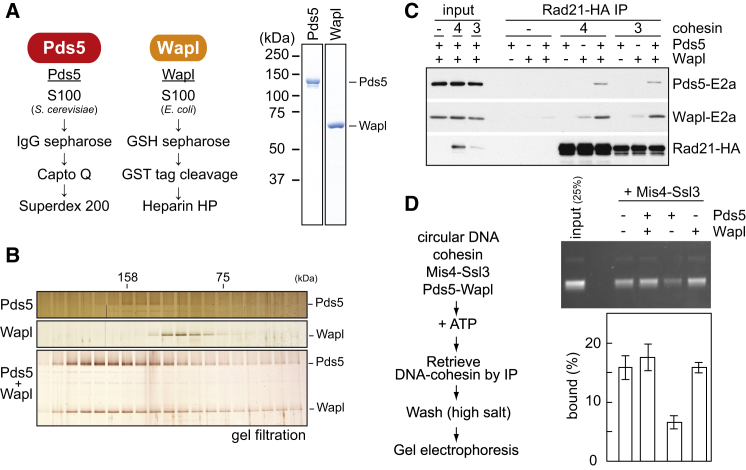
Fission Yeast Pds5 and Wapl and Their Effect on Cohesin Loading (A) Purification schemes for recombinant Pds5 and Wapl and analysis of the purified proteins by SDS-PAGE and Coomassie blue staining. (B) Pds5 forms a complex with Wapl. Purified Pds5 and Wapl were mixed and analyzed by gel filtration. Fractions were analyzed by SDS-PAGE, and proteins visualized by silver staining. The elution positions of two size markers (aldolase and conalbumin) are indicated. (C) Pds5 and Wapl interact with the cohesin complex. Recombinant cohesin tetramer (Psm1, Psm3, Rad21, and Psc3, “4”) or trimer (Psm1, Psm3, and Rad21, “3”) complexes were immunoprecipitated in the presence or absence of Pds5 and/or Wapl. Bound proteins were analyzed by western blotting. 1/20 of the input and Rad21-HA immunoprecipitates are shown. (D) Wapl stimulates loading of the cohesin holocomplex by Mis4-Ssl3. Schematic of the in vitro cohesin-loading assay and a gel image of the recovered DNA. All reactions contained cohesin, Mis4-Ssl3, plus the indicated proteins and were incubated for 15 min. The graph shows means and standard deviations from at least three independent experiments. See also Figure S1 for an analysis of how Pds5 counteracts loading.

A purified fission-yeast cohesin tetramer, consisting of a Psm1-Psm3 dimer, Rad21, and Psc3, topologically loads onto DNA in vitro in a reaction that is facilitated by the Mis4-Ssl3 cohesin loader (Murayama and Uhlmann, 2014). Two additional cohesin subunits, Pds5 and Wapl, have been implicated in regulating cohesin’s chromosome association in vivo. To characterize their activities, we purified Pds5 and Wapl following overexpression in budding yeast and *E. coli*, respectively (Figure 1A). Combining equimolar amounts of Pds5 and Wapl led to formation of a stable heterodimer complex (Figure 1B).

We examined Pds5 and Wapl association with the cohesin complex by co-immunoprecipitation. Pull-down of the cohesin tetramer led to co-purification of Pds5 and Wapl, if they were both added (Figure 1C). The cohesin tetramer showed a weaker interaction with Wapl and hardly detectable binding to Pds5 on their own. This suggests that Pds5 and Wapl coordinately bind to the cohesin tetramer. Budding yeast cells contain substoichiometric amounts of Wapl, compared to Pds5 (Chan et al., 2012). Moreover, Wapl, but not Pds5, is dispensable for cell viability. Therefore, whereas Pds5 and Wapl jointly act in cohesin holocomplex formation, Pds5 must be able to fulfill part of its role independently of Wapl. Pds5 and Wapl associated with a cohesin trimer, containing Psm1-Psm3 and Rad21 but lacking Psc3, with equal efficiency compared to the cohesin tetramer (Figure 1C). This implies that Pds5 and Wapl make extensive contacts with cohesin, in addition to the reported interaction of Wapl with a human Psc3 ortholog (Hara et al., 2014).

**Figure S1:**
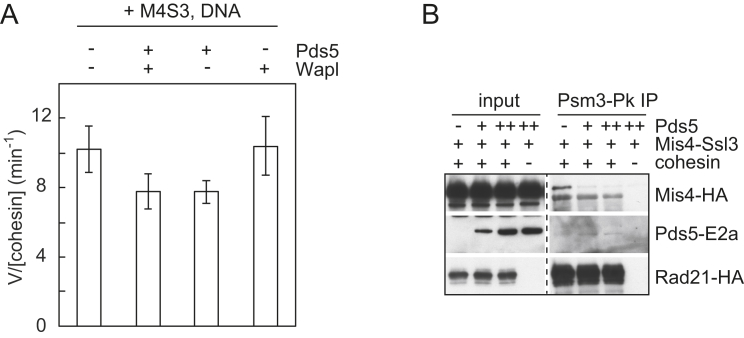
Pds5 Antagonizes Mis4-Ssl3 Interaction with Cohesin, Related to Figure 1 (A) Cohesin’s ATPase activity was monitored in the presence of Mis4-Ssl3, DNA, and the indicated proteins. Shown are the means and standard deviations from three independent experiments. (B) Co-immunoprecipitation of cohesin (30 nM) and Mis4-Ssl3 (50 nM) were analyzed in the presence of additional “+,” 50 nM and “++,” 100 nM Pds5 protein.

We next investigated the impact of Pds5 and Wapl on topological cohesin loading onto a circular DNA substrate. Following incubation in the presence of the cohesin loader and ATP, cohesin is retrieved by immunoprecipitation of Psm3 and bound DNA quantified by gel electrophoresis (Murayama and Uhlmann, 2014). To our surprise, Pds5 addition to the cohesin tetramer markedly inhibited the loading reaction (Figure 1D). Despite not forming a stable complex with cohesin, Pds5 dampened cohesin’s ATPase activity and competed with Mis4-Ssl3 for cohesin binding (Figures S1A and S1B). Strikingly, addition of Wapl to the loading reaction compensated for Pds5’s inhibitory effect and restored loading to levels equal if not greater than that of the cohesin tetramer (Figure 1D). Wapl did not stimulate ATP hydrolysis (Figure S1A), suggesting that it facilitates DNA binding in a different way. Thus, within the cohesin holocomplex that includes Pds5, Wapl facilitates cohesin loading onto DNA. This is at first sight unexpected, as Wapl was thought of as a cohesin unloader. However, we note that budding yeast cells lacking Wapl display reduced chromatin-bound cohesin levels (Rowland et al., 2009, Sutani et al., 2009), consistent with a role of Wapl in cohesin loading.

### Cohesin Unloading from DNA by Pds5 and Wapl

**Figure 2:**
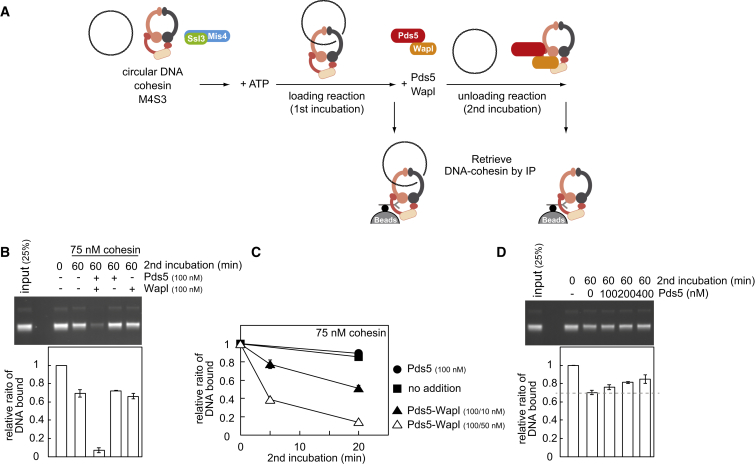
Pds5-Wapl Promotes Cohesin Unloading from DNA (A) Schematic of the in vitro cohesin-unloading assay. (B) The gel and graph show DNA recovered before (0 min) and after (60 min) the second unloading incubation, in which the indicated proteins were included. See also Figures S2A–S2C for indication that DNA and protein remained intact during the incubation. (C) Time-course analysis of the unloading reaction, including the indicated protein concentrations. (D) Pds5 counteracts spontaneous cohesin unloading. In vitro unloading reactions were carried out in the presence of the indicated concentrations of Pds5. All graphs show means and standard deviations from at least three independent experiments. See also Figure S2D, which shows that Mis4-Ssl3 has no detectable role during unloading.

**Figure S2:**
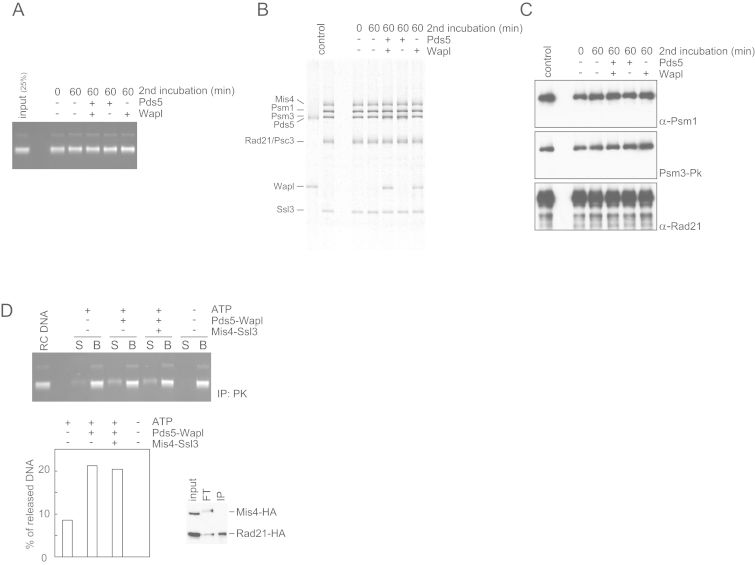
Characterization of Pds5-Wapl-Mediated Cohesin Unloading, Related to Figure 2 (A–C) The total DNA contained in the reaction was visualized by agarose-gel electrophoresis (A), and cohesin subunits were analyzed by SDS-PAGE and silver staining or western blotting, respectively, following the cohesin-unloading incubation (B and C). This confirmed that all macromolecular components remained intact. (D) Mis4-Ssl3 is dispensable for Pds5- and Wapl-dependent cohesin unloading. Cohesin-DNA complexes were recovered by anti-Pk-immunoprecipitation of the Psm3 subunit. Western blotting revealed that Mis4-Ssl3 is not part of the cohesin-DNA complex retrieved by immunoprecipitation. Then a DNA release assay was carried out in the presence of the indicated components (100 nM Psc3, with or without 400 nM of Pds5 and Wapl and 100 nM Mis4-Ssl3). DNA released into the supernatant (“S”) is shown next to the DNA that remained bound to the beads (“B”), the percentage of released DNA was quantified.

Wapl is thought of as a cohesin unloader that facilitates dynamic cohesin turnover on chromosomes (Bernard et al., 2008, Kueng et al., 2006, Lopez-Serra et al., 2013). We therefore used the following reaction scheme to analyze the cohesin-unloading activity of Wapl (Figure 2A). First, cohesin tetramers were topologically loaded onto DNA, aided by Mis4-Ssl3, in an incubation at low ionic strength (buffer including 30 mM NaCl). This was followed by a second incubation at increased salt concentration (buffer including 150 mM NaCl), when Pds5 and Wapl were added. Cohesin was immunopurified after the first or second incubation and cohesin-bound DNA quantified. If the second incubation lacked additional protein, we observed a small but reproducible loss of DNA, which we interpret as spontaneous DNA unloading (Figure 2B). Addition of Wapl alone did not affect unloading, but addition of Wapl together with Pds5 led to almost complete loss of DNA. Titrating the Wapl concentration showed that even substoichiometric amounts of Wapl efficiently dissociated cohesin from DNA (Figure 2C). The DNA and all the constituent proteins remained intact during these incubations (Figures S2A–S2C), indicating that unloading was not due to nuclease or protease contamination. This suggests that, as well as facilitating DNA entry, Wapl is indeed a potent catalyst of DNA exit from the cohesin ring.

Addition of increasing concentrations of Pds5, in the absence of Wapl, protected cohesin from spontaneous unloading (Figure 2D). This mirrors the situation during cohesin loading and suggests that Pds5 stabilizes the cohesin ring and counteracts DNA entry or exit.

The above unloading reactions were performed in solution, before cohesin was retrieved to analyze bound DNA. We also performed a reaction in which cohesin-DNA complexes were immunopurified after the loading reaction, followed by high-salt washes to remove the Mis4-Ssl3 cohesin loader and other components of the loading reaction. Addition of Pds5 and Wapl, while cohesin remained immobilized on beads, reproduced DNA unloading albeit with lower efficiency (Figure S2D). This setup allowed us to address whether the cohesin loader and ATP play a role in cohesin unloading, as they do during loading. We could not detect a role for Mis4-Ssl3; however, unloading depended on the presence of ATP.

### ATP Hydrolysis Dependence of Cohesin Unloading

**Figure 3:**
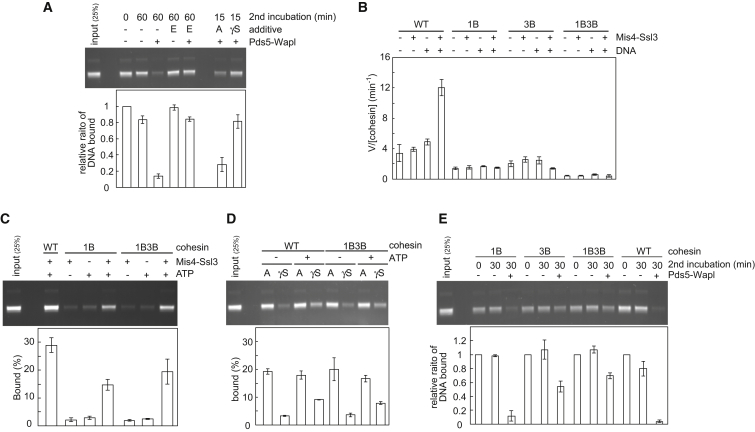
ATP Hydrolysis Dependence of Cohesin Unloading (A) Cohesin-unloading reactions were performed in the presence of the indicated compounds: “E,” EDTA; “A,” ATP; “γS,” ATP-γ-S. The graph shows means and standard deviations from three independent experiments. (B) Effect of the Walker B mutations on the cohesin ATPase. The indicated cohesin tetramer complexes were incubated with or without Mis4-Ssl3 and RC-DNA. ATP hydrolysis was assayed and quantified by thin-layer chromatography. “1B” and “3B” denote Psm1(E1161Q) and Psm3(E1128Q) mutant cohesin, respectively. (C) Walker B mutant cohesin complexes are DNA-loading proficient. Loading assays were performed and quantified with the indicated cohesin complexes and cofactors. Means and standard deviations from three independent experiments are shown. See also Figures S3A–S3C for the various purified cohesin complexes, a time course of loading, and confirmation of the topological nature of DNA binding by Walker B mutant cohesin. (D) Walker B mutant cohesin loading remains ATP hydrolysis dependent. The loading assays were carried out with or without ATP in the presence of additional ATP (“A”) or ATPγS (“γS”). The quantification denotes mean and errors derived from two experiments. Addition of ATPγS on top of ATP was used to confirm that ATPγS does in fact bind to and inhibit the SMC ATPase. (E) Walker B mutant cohesin is unloading defective. The indicated cohesin complexes were loaded onto DNA (time “0”), and comparable amounts used as substrate for a 30 min unloading incubation with or without Pds5-Wapl. Shown are means and standard deviations from three independent experiments. See also Figures S3D–S3F for the differential effect of divalent cations on cohesin’s ATPase, loading and unloading.

Chelating divalent Mg^2+^ ions by EDTA blocks ATP-dependent reactions. Addition of EDTA after cohesin loading, but before the unloading incubation, abolished cohesin unloading by Pds5-Wapl (Figure 3A). It also blocked the low level of spontaneous, Pds5-Wapl-independent cohesin unloading during the second incubation. This suggests that the topologically DNA-bound cohesin tetramer spontaneously, but inefficiently, unloads from the DNA in an ATP-dependent manner, a reaction that is greatly stimulated by Pds5-Wapl. To confirm the specificity of the EDTA effect and to determine whether ATP must be hydrolyzed for cohesin unloading, we utilized unhydrolyzable ATP-γ-S. Its addition to the unloading reaction, but not additional ATP, blocked cohesin unloading from DNA by Pds5-Wapl (Figure 3A). This suggests that, similar to DNA entry, DNA exit out of the cohesin ring requires ATP hydrolysis.

### Differential ATPase Requirements for DNA Entry and Exit

**Figure S3:**
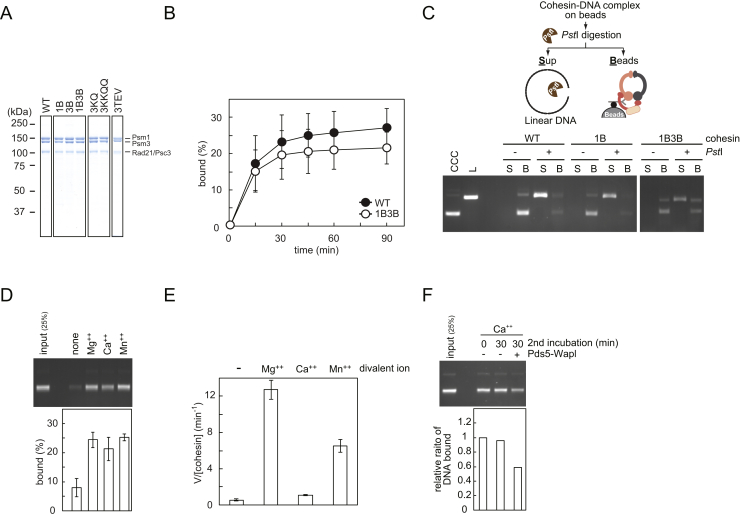
The Role of ATP Hydrolysis for Cohesin Loading and Unloading, Related to Figure 3 (A) Purifications of wild-type and Walker B mutant (1B, 3B, or 1B3B), as well as lysine 105 and 106 mutant (3KQ or 3KKQQ) cohesin tetramer complexes and a cohesin tetramer containing TEV protease cleavable Psm3 (3TEV). Proteins from the final gel-filtration step were analyzed by SDS-PAGE and Coomassie blue staining. (B) Time course of the cohesin-loading reaction, comparing wild-type and double Walker B mutant (1B3B) cohesin complexes. (C) Confirmation that Walker B mutant cohesin complexes topologically entrap DNA. A schematic of the reaction is shown. DNA-cohesin complexes were immobilized on magnetic beads, then bound cccDNA was linearized by restriction enzyme PstI treatment, which cuts once in pBluescript. Release of linear DNA into the supernatant (“S”) or its retention on the beads (“B”) was analyzed by gel electrophoresis. The gel image shows a typical result of the experiment. (D) Little impact of different divalent cations on cohesin loading. Cohesin-loading reactions were carried out with the indicated divalent cations (1 mM) and Mis4-Ssl3. (E) Inhibition of the cohesin ATPase by Ca^2+^ or Mn^2+^. The effect of the indicated divalent cations on cohesin’s ATPase activity, in the presence of Mis4-Ssl3 and DNA, was measured. Panels (B), (D), and (E) report means and standard deviations from three independent experiments. (F) Ca^2+^ inhibits cohesin unloading by Pds5 and Wapl. A cohesin-unloading reaction was carried out in the presence of Ca^2+^ instead of Mg^2+^.

To corroborate that ATP hydrolysis is required for cohesin unloading, we analyzed the behavior of cohesin complexes carrying glutamate-to-glutamine changes in the Walker B motif of the SMC ATPase. In the cases of *P. furiosus* SMC and budding yeast cohesin, these mutations substantially slow down ATP hydrolysis and stabilize ATP-dependent SMC head dimerization (Arumugam et al., 2006, Hu et al., 2011, Lammens et al., 2004). We purified fission-yeast cohesin tetramers containing Psm1(E1161Q) “1B,” Psm3(E1128Q) “3B,” or both mutations “1B3B” (Figure S3A). As expected, the rate of ATP hydrolysis by these complexes was substantially reduced, compared to wild-type tetramers (Figure 3B). To our surprise, in vitro loading of cohesin onto DNA was less severely affected. Even 1B3B mutant cohesin complexes retained over half of the topological loading potential of the wild-type complex (Figures 3C, S3B, and S3C). Nonetheless, loading onto DNA of these mutant complexes remained ATP hydrolysis dependent and was inhibited by addition of ATP-γ-S (Figure 3D). This suggests that ATP hydrolysis is not rate limiting for cohesin loading. Rather, adoption of a conformational change, promoted by the cohesin loader, might limit cohesin loading (Murayama and Uhlmann, 2014). The conformational change in turn might induce ATP hydrolysis, even if ATPase function is compromised. We came to a similar conclusion from comparing different divalent cations for their ability to support cohesin loading. Replacing Mg^2+^ with Ca^2+^ or Mn^2+^ in the reaction buffer greatly attenuated ATP hydrolysis by cohesin, but to a much lesser degree its loading onto DNA (Figures S3D and S3E).

We were now able to prepare comparable amounts of wild-type, 1B, 3B, and 1B3B cohesin-DNA complexes as substrates for cohesin unloading. Whereas 1B cohesin was only mildly affected, 3B cohesin and even more so 1B3B cohesin were greatly defective in unloading by Pds5-Wapl (Figure 3E). This suggests that Walker B motif-dependent ATP hydrolysis is important for DNA exit from the cohesin ring and that ATP hydrolysis limits unloading to a greater degree than the loading reaction. Consistently, Ca^2+^, which could replace Mg^2+^ during loading, only inefficiently supported unloading (Figure S3F). Taken together, these results show that both DNA entry into and exit out of the cohesin ring are ATP hydrolysis-dependent reactions. Both reactions therefore likely involve disengagement of the SMC ATPase heads.

### Wapl Opens the Rad21N-Psm3 Interface

**Figure 4:**
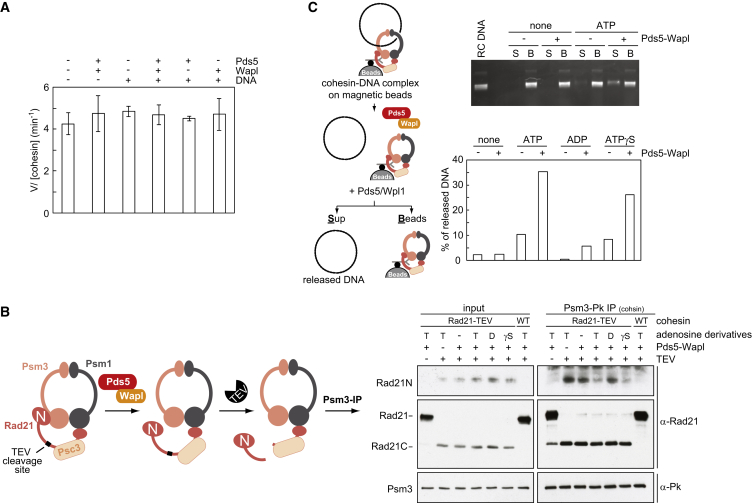
Wapl Opens the Rad21N-Psm3 Ring Interface (A) Pds5 and Wapl do not affect the cohesin ATPase. ATP hydrolysis was monitored in the presence of the indicated proteins and RC-DNA. Pds5 and Wapl by themselves did not show detectable ATP hydrolysis (data not shown). Means and standard deviations from three independent experiments are shown. (B) Schematic and results of the “Rad21N dissociation” experiment. Cohesin carrying TEV-cleavable Rad21, or wild-type cohesin as control, was incubated with Pds5-Wapl, in presence or absence of ATP (T), ADP (D), or ATP-γ-S (γS), then Rad21 was cut by TEV protease. The resultant cohesin was recovered by immunoprecipitation, and the Rad21 subunit and its two cleavage products were monitored by western blotting. See also Figure S4 for the full image of the western blot, an experiment examining the activity of Mis4-Ssl3 in this assay, and demonstration of DNA binding by Pds5, Wapl, and Psc3. (C) Schematic and results of the “DNA release” experiment in which cohesin-DNA complexes are first isolated on magnetic beads. The unloading reaction was then initiated in the presence of the indicated components. The gel image shows the supernatant “S” and bead “B” fractions following the unloading incubation. The percentage of released DNA was quantified.

How does Wapl facilitate both DNA entry into as well as DNA exit out of the cohesin ring? Mis4-Ssl3 stimulates ATP hydrolysis during DNA entry (Murayama and Uhlmann, 2014); however, the rate of ATP hydrolysis by a cohesin tetramer was not increased by Pds5-Wapl under conditions of both cohesin loading (Figure S1A) or unloading (Figure 4A).

**Figure S4:**
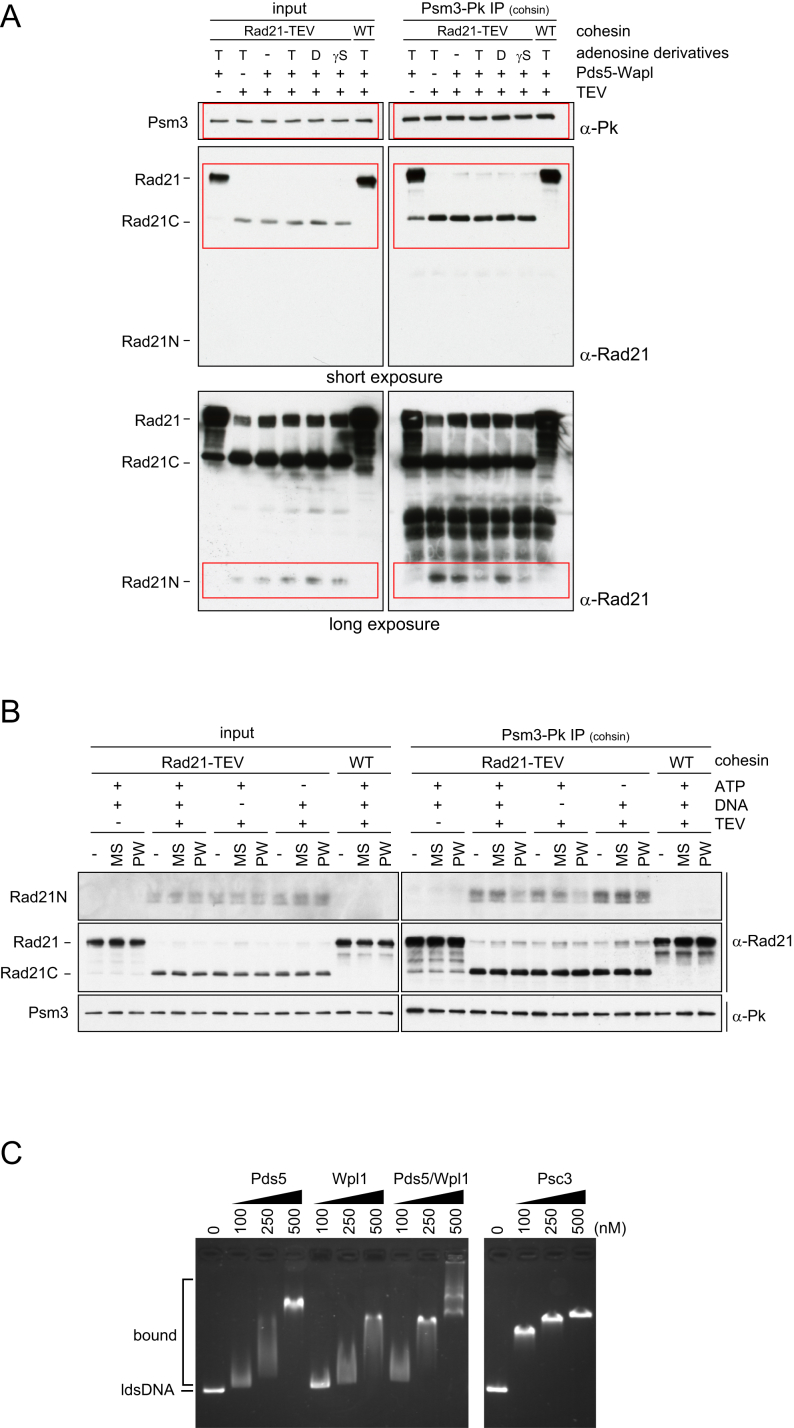
Pds5-Wapl Facilitates Psm3-Rad21 Interface Opening, Related to Figure 4 (A) The original western blot images from Figure 4B. Red rectangles denote the selected areas. (B) Comparison of Pds5-Wapl and Mis4-Ssl3 in the Rad21-N dissociation assay. Cohesin carrying TEV-cleavable Rad21 was incubated with Mis4-Ssl3 (“MS”) or Pds5-Wapl (“PW”), in presence or absence of DNA and/or ATP, then Rad21 was cut by TEV protease. The resultant cohesin was recovered by immunoprecipitation and the Rad21 subunit and its two cleavage products were monitored by western blotting. (C) Pds5 and Wapl are DNA-binding proteins. The indicated concentrations of Pds5 and/or Wapl were mixed with a 3.0 kb linear double-stranded DNA (lds DNA) substrate and incubated at 32°C for 10 min. Psc3 was similarly analyzed. The DNA-protein complexes were analyzed by 0.8% agarose gel electrophoresis.

It has been suggested that Wapl facilitates cohesin dissociation from chromosomes by opening a DNA exit gate between the kleisin N terminus and Smc3 (Chan et al., 2012, Huis in ’t Veld et al., 2014). We therefore tested whether Wapl indeed causes dissociation of the Rad21 N terminus from fission yeast Psm3. We used purified cohesin tetramers in which one of the two separase cleavage sites in Rad21 was changed for the recognition sequence of TEV protease (Murayama and Uhlmann, 2014, Tomonaga et al., 2000). Following TEV cleavage, both the N- and C-terminal cleavage products (Rad21N and Rad21C, respectively) remained associated with the cohesin tetramer, which was immunopurified from the reaction via an epitope tag on Psm3. Addition of Pds5-Wapl resulted in loss of Rad21N, but not Rad21C, from the cohesin complex, demonstrating that Pds5-Wapl indeed displaces the kleisin N terminus (Figures 4B and S4A). Rad21N displacement occurred in the presence of ATP or ATP-γ-S, but not in the presence of ADP or in the absence of nucleotide. In contrast, Mis4-Ssl3 in the presence or absence of ATP and DNA did not achieve Rad21N displacement (Figure S4B). These results confirm that Pds5-Wapl opens the cohesin ring interface between Rad21N and Psm3. The requirement for ATP, but not its hydrolysis, further suggests that the Rad21N-Psm3 interface only opens if the SMC head dimer is occupied by ATP and therefore closed.

We also tested the ability of Pds5 and Wapl to contact DNA. A gel-mobility shift showed that Pds5, Wapl, and also Psc3, which we included in this analysis, associate with DNA (Figure S4C). This is reminiscent of DNA binding by most of the non-SMC components of the budding-yeast condensin complex (Piazza et al., 2014). It suggests that several of cohesin’s subunits engage in electrostatic contacts with DNA that might help to shape the DNA path during the entry and exit reactions.

Given that ATP, but not its hydrolysis, was required for Wapl-dependent Rad21N displacement, we revisited the nucleotide requirements during cohesin unloading. Cohesin unloading in our sequential two-step loading and unloading assay was efficiently blocked by non-hydrolyzable ATP (Figure 3A). However, if we immunopurified cohesin-DNA complexes following the loading reaction, including an overnight affinity-capture step in the absence of nucleotide, then ATP-γ-S permitted Pds5-Wapl-stimulated unloading almost as efficiently as ATP (Figure 4C). Little unloading was observed with ADP or without nucleotide. This suggests that ATP hydrolysis promotes an early step during DNA exit from the cohesin ring that can in part be replaced by a longer incubation without nucleotide, possibly corresponding to SMC head disengagement. After this, DNA exit remains dependent on the presence of ATP, but not its hydrolysis, consistent with the characteristics of Rad21N disengagement.

### Wapl Is Both a Cohesin Loader and Unloader

**Figure 5:**
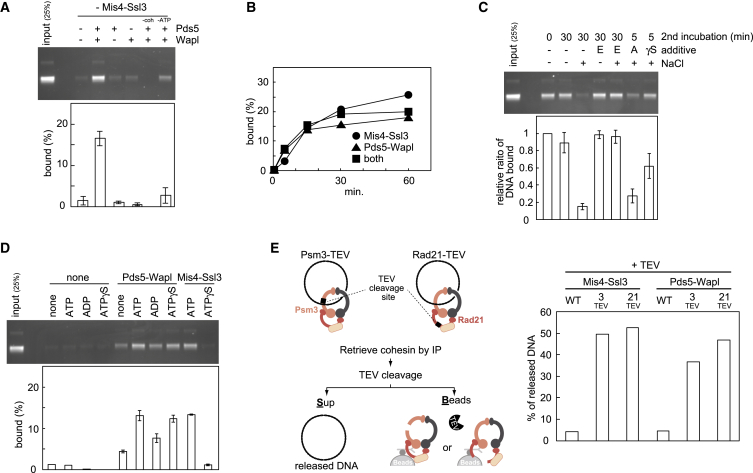
Pds5-Wapl Is both Loader and Unloader (A) Cohesin-loading reactions (15 min) were carried out without the Mis4-Ssl3 cohesin loader, but including Pds5-Wapl. Reactions omitting cohesin or ATP are shown as controls. See also Figure S5A for confirmation of topological cohesin loading by Pds5-Wapl. (B) Comparison of cohesin loading over time in the presence of Mis4-Ssl3, Pds5-Wapl, or both. See also Figures S5B–S5D for titration of Mis4-Ssl3 and Pds5-Wapl and illustration that Pds5-Wapl-dependent reactions require Psc3. (C) Following loading by Pds5 and Wapl for 30 min, cohesin unloading was initiated by increasing the salt concentration to 150 mM NaCl in the presence of the indicated compounds: “E,” EDTA; “A,” ATP; “γS,” ATPγS. (D) Cohesin-loading reactions were performed for 15 min with indicated loaders and adenosine derivatives. (E) Schematic and quantification of DNA released from cohesin rings following TEV-protease cleavage of either Psm3 (3 TEV) or Rad21 (21 TEV) after cohesin had been loaded by either Mis4-Ssl3 or Pds5-Wapl. See also Figures S5E–S5G for controls for loading and cleavage of the two TEV-cleavable cohesin complexes.

**Figure S5:**
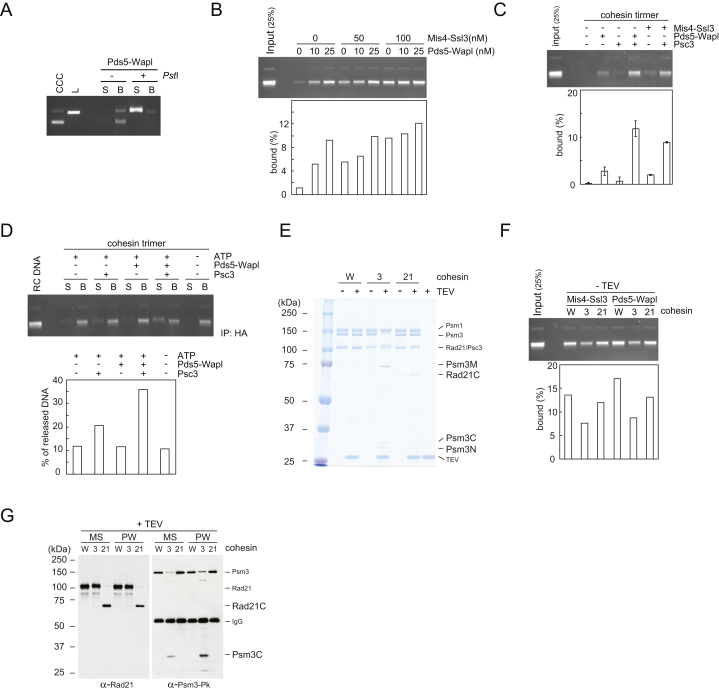
Control Experiments for Pds5-Wapl-Dependent Cohesin Loading, Related to Figure 5 (A) Confirmation of topological cohesin loading by Pds5-Wapl. Cohesin-loading reactions were carried out with Pds5-Wapl for 15 min, then bound DNA was digested and analyzed as described in Figure S3C. (B) Stimulation of cohesin loading by Pds5-Wapl. Cohesin-loading reactions were carried out including the indicated concentrations of Mis4-Ssl3 and Pds5-Wapl. The band intensities were quantified. (C and D) Psc3 is required for both Pds5- and Wapl-dependent cohesin loading and unloading, respectively. The DNA loading and release assays were carried out using a purified cohesin trimer (Murayama and Uhlmann, 2014), devoid of the Psc3 subunit, supplemented with the indicated components. Means and standard deviations from three independent experiments are shown; the reactions with Mis4-Ssl3 were included twice. (E) TEV-protease cleavage of Psm3-TEV (3) and Rad21-TEV (21) cohesin complexes. The various cohesin complexes were incubated with TEV protease at 16°C for 1.5 hr, then analyzed by SDS-PAGE and Coomassie staining. The wild-type cohesin tetramer (W) served as a comparison. A size marker is shown and the band identities are indicated (N, M, and C indicate N-terminal, middle, and C-terminal protein fragments, respectively). (F) Confirmation of comparable loading of the cohesin complexes shown in (E) onto DNA using Mis4-Ssl3 or Pds5-Wapl. (G) Western blot analysis of Rad21 and Psm3 cleavage during the experiment shown in Figure 5E. Aliquots (0.5 μl out of 15 μl) of the immunoprecipitate were analyzed by SDS-PAGE followed by western blotting against Rad21 or against the C-terminal Pk epitope on Psm3. Fragment annotation is as in (E).

We next addressed whether Wapl’s ability to promote cohesin loading depends on the presence of Mis4-Ssl3, or whether Wapl can act independently as a cohesin loader. Addition of Pds5-Wapl to loading reactions greatly promoted topological cohesin loading, even in the absence of Mis4-Ssl3 (Figures 5A and S5A). When we compared the efficiencies of loading, Mis4-Ssl3 performed best, followed closely by Pds5-Wapl. Addition of Pds5-Wapl on top of the cohesin loader did not further increase loading (Figure 5B). Even at low Mis4-Ssl3 concentrations, Pds5-Wapl augmented loading only by a small margin (Figure S5B). These results suggest that cohesin loading is independently promoted by Mis4-Ssl3 or Pds5-Wapl. Both protein complexes appear to act on one interlinked reaction path, such that addition of both hardly exceeds the effect of either alone.

In common with Mis4-Ssl3-stimulated loading (Murayama and Uhlmann, 2014), cohesin loading (as well as unloading) by Pds5-Wapl depended on the Psc3 subunit (Figures S5C and S5D). This was the case even though Psc3 is not required for Pds5-Wapl to interact with cohesin (Figure 1C). This is consistent with the possibility that a common reaction, independently stimulated by the cohesin loader or by Pds5-Wapl, facilitates DNA entry and exit.

If Pds5-Wapl facilitates both DNA entry into and exit and out of the cohesin ring, what determines the directionality of the reaction? Our loading reactions are performed at lower salt concentrations, whereas unloading occurs at increased ionic strength. We therefore tested whether the buffer conditions alone can change the directionality of DNA transport. To this end, we incubated cohesin tetramers under loading conditions with Pds5-Wapl, resulting in cohesin loading as before. Increasing the salt concentration in the reaction was sufficient to reverse the reaction, leading to loss of cohesin from DNA (Figure 5C). Addition of EDTA or ATP-γ-S in the second incubation inhibited unloading, confirming that it remains an ATP hydrolysis-dependent enzymatic reaction under these conditions. These findings illustrate the dynamic behavior of cohesin and suggest that a subtle difference, possibly in the conformation of the complex that under our conditions is influenced by the ionic strength of the incubation buffer, changes the directionality of DNA transport. Other regulators, e.g., the cohesin loader, might impact on this equilibrium in their own way.

### DNA Entry into Cohesin’s Central Topological Cavity

When we analyzed the nucleotide requirements of Pds5-Wapl-catalyzed cohesin loading, we found that non-hydrolyzable ATP-γ-S was almost as effective as ATP. This contrasts with Mis4-Ssl3-catalyzed loading, which requires ATP hydrolysis (Figure 5D). It is instead reminiscent of Rad21-N displacement from Psm3 by Pds5-Wapl, which requires ATP but not its hydrolysis. Based on these considerations, we envision two scenarios for how Pds5-Wapl could promote cohesin loading. Opening the Rad21-N interface could allow DNA entry into a gap between Rad21 and the SMC heads. The resulting entrapment between kleisin and SMC heads would be topological, even though the SMC heads would not open and DNA would not actually reach the center of the ring. In this scenario, loading would be incomplete and maybe unphysiological. Alternatively, Pds5-Wapl stimulates the same reaction trajectory as Mis4-Ssl3, while emphasizing a second step that requires ATP but not its hydrolysis. Head disengagement might be promoted during this reaction by a conformational change that Pds5-Wapl imposes. Note that ATP-γ-S is an imperfect ATP mimic and poorly sustains SMC head dimerization (Hu et al., 2011).

To distinguish between the two scenarios, we asked whether DNA reaches cohesin’s central cavity, following loading by either Mis4-Ssl3 or Pds5-Wapl. To do this, we inserted two TEV protease recognition sites on opposite strands of the Psm3 coiled coil (Gruber et al., 2006). Psm3 cleavage opens the cohesin ring without disrupting kleisin interactions with the SMC heads (Figures S5E–S5G). As comparison we used TEV-cleavable Rad21. Following cleavage, the C-terminal kleisin fragment disrupts the SMC head interaction (Weitzer et al., 2003), such that both the central ring as well as a possible kleisin trap are disrupted. Both cohesin variants were loaded onto DNA with comparable efficiency to wild-type complexes by either Mis4-Ssl3 or Pds5-Wapl (Figure S5F). The resulting cohesin-DNA complexes were immobilized on beads and treated with TEV protease. This resulted in efficient DNA release from cohesin that contained TEV protease recognition sites in Psm3 or Rad21, but not from wild-type cohesin, irrespective of whether cohesin had been loaded onto DNA by Mis4-Ssl3 or Pds5-Wapl (Figure 5E). These results suggest that both loaders catalyze complete DNA entry into the cohesin ring. The two complexes might catalyze their respective part of a concerted loading reaction that requires disengagement of both the ATPase heads and the Rad21N-Psm3 interface.

### Psm3 Acetylation Sites Control DNA-Dependent ATP Hydrolysis

**Figure 6:**
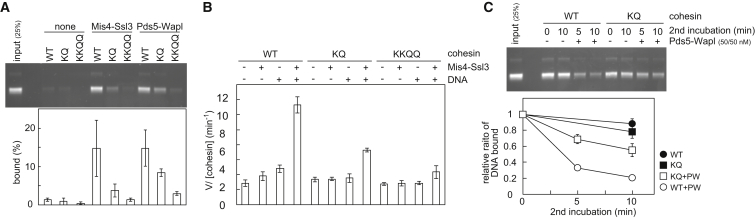
Psm3 Acetylation Acceptor Lysines are DNA Sensors during Loading and Unloading (A) Cohesin-loading reactions were performed using wild-type (WT), Psm3K106Q (KQ), and Psm3K105Q,K106Q (KKQQ) cohesin tetramer complexes in the presence of the indicated loading factors. See also Figure S3A for the purification of the mutant cohesin complexes. (B) ATPase activity of the indicated cohesin tetramers with or without added Mis4-Ssl3 and/or DNA. (C) Wild-type and KQ cohesin was loaded onto DNA, and equivalent concentrations of cohesin-DNA complexes were used as substrate in unloading reactions initiated by Pds5-Wapl (PW). Retained DNA was quantified over time; the means and standard deviations from three independent experiments are shown.

Smc3 acetylation during S phase generates a stably chromosome-bound cohesin pool that promotes enduring sister-chromatid cohesion. To understand the consequence of Smc3 acetylation, we purified fission-yeast cohesin tetramers in which lysine K106 or both lysines K105 and K106, which act as the acetylation acceptors, were replaced with glutamine (denoted KQ and KKQQ, respectively; Figure S3A). When we used these cohesin complexes in our in vitro loading assays, we found that DNA loading was greatly reduced by the KQ mutation and obliterated by the KKQQ change in a loading reaction catalyzed by Mis4-Ssl3 or by Pds5-Wapl (Figure 6A). This suggests that the two conserved lysines on the Psm3 head make a critical contribution to the DNA-entry reaction. This is consistent with the observation that budding-yeast cohesin bearing equivalent KKQQ mutations barely associates with chromosomes in vivo (Hu et al., 2015).

A recently reported crystal structure of the Rad50 SMC head dimer, in complex with DNA, shows DNA binding to a surface loop rich in lysines, equivalent to the Smc3/Psm3 surface loop from which K105 and K106 emanate (Gligoris et al., 2014, Rojowska et al., 2014). We therefore investigated the possibility that K105 and K106 act as DNA sensor during the loading reaction. We first compared DNA-stimulated ATP hydrolysis between wild-type, KQ, and KKQQ cohesin. The basal, DNA-independent ATPase activity of all three complexes was comparable. However, DNA stimulation of the ATPase was reduced in the case of KQ cohesin and undetectable in the KKQQ mutant (Figure 6B). This suggests that DNA contact with K105 and K106 stimulates ATP hydrolysis and thereby the DNA-entry reaction.

The ability of KQ cohesin to load onto DNA, albeit at a reduced rate, allowed us to assess the effect of this mutation on cohesin unloading from DNA. We prepared equal amounts of wild-type and KQ mutant cohesin-DNA complexes and used them as substrate in an unloading reaction. This showed that the KQ mutant was markedly compromised in unloading from DNA (Figure 6C). We could not test the effect of the KKQQ mutation on unloading as we could not load this complex onto DNA. Together, this suggests that the conserved lysines on the Psm3 head act as a DNA sensor to trigger ATP hydrolysis and that this is essential for both DNA entry into and exit out of the cohesin ring.

### An SMC-Hinge Interaction with the Cohesin Loader and with Psc3

Our previous results, based on peptide-array interactions, suggested that the cohesin loader makes multiple contacts with cohesin around its ring circumference, including Psc3 and the SMC hinge (Murayama and Uhlmann, 2014). Structural analysis of the cohesin loader in turn shows that its dimensions are insufficient to span the cohesin ring diameter (Chao et al., 2015). If the cohesin loader indeed contacts both Psc3 and the SMC hinge, and if these contacts occur simultaneously, the ring will have to undergo a substantial conformational change to accommodate these interactions.

**Figure 7:**
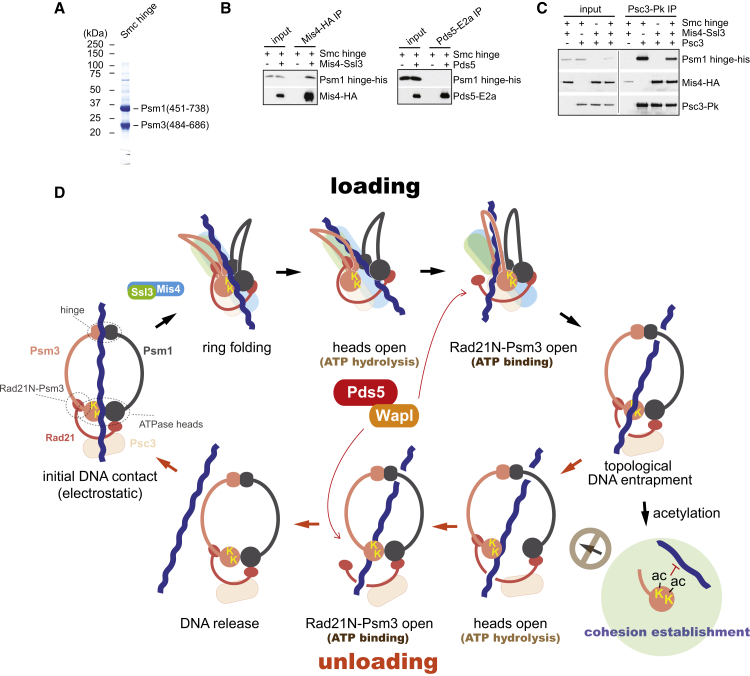
Cohesin Hinge Interactions and a Unified Model for Cohesin Loading and Unloading (A) Purification of a Psm1-Psm3 hinge dimer. See also Figure S6A, which shows DNA binding by the Psm1-Psm3 hinge. (B) The Psm1-Psm3 hinge interacts with Mis4-Ssl3. Co-immunoprecipitation of the cohesin loader, or Pds5 as control, with the cohesin hinge was analyzed by western blotting. (C) A supramolecular complex between the Psm1-Psm3 hinge, Psc3, and the cohesin loader. Psc3 was immunoprecipitated, and co-precipitation of the cohesin hinge and the cohesin loader were analyzed. (D) A unified model for DNA entry and exit into and out of the cohesin ring. See Discussion for details. See also Figure S6B for alternative models for DNA entry into the cohesin ring.

**Figure S6:**
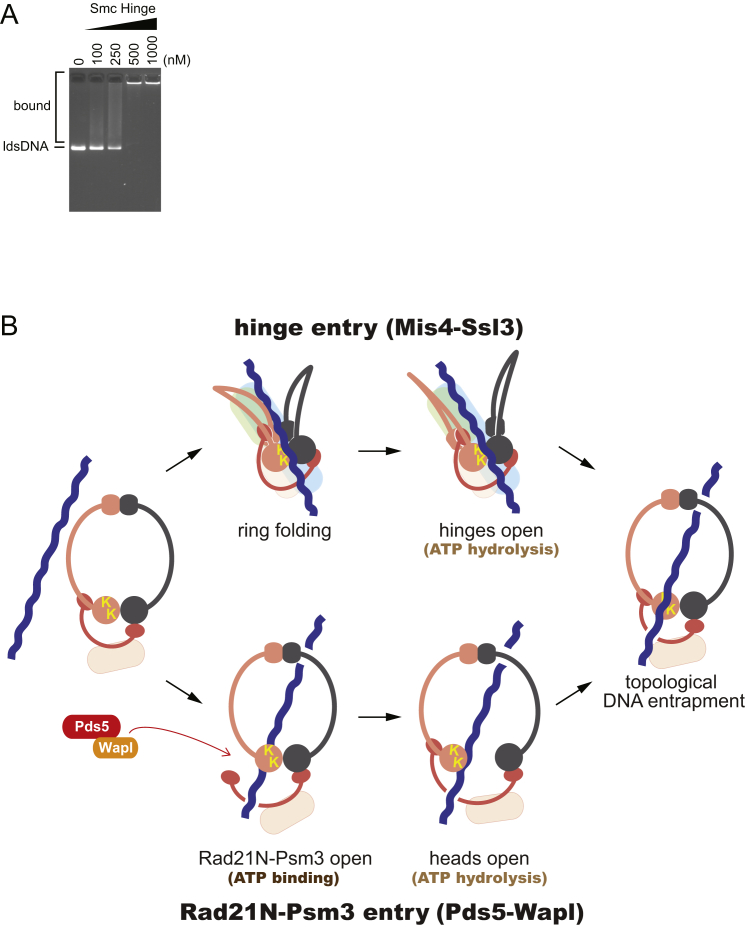
DNA Binding by the Psm1-Psm3 Hinge and Alternative Possibilities for DNA Entry Mediated by Mis4-Ssl3 or Pds5-Wapl, Related to Figure 7 (A) The Psm1-Psm3 hinge contains DNA-binding activity. The indicated concentrations of Psm1-Psm3 hinge were mixed with a 3.0 kb linear double-stranded DNA (lds DNA) substrate and incubated at 32°C for 10 min. The DNA-protein complexes were analyzed by 0.8% agarose-gel electrophoresis. (B) Alternative possibilities for DNA entry mediated by Mis4-Ssl3 or Pds5-Wapl. See the Discussion in the main text for details.

To investigate the possibility of direct cohesin-hinge interactions, we purified a recombinant Psm1-Psm3 hinge dimer. The constructs included short coiled-coil regions to span areas that were suggested by the peptide-array results to be loader interaction sites (Figure 7A). Indeed, the Psm1-Psm3 hinge directly interacted with the Mis4-Ssl3 cohesin loader. The interaction was specific, as the hinge did not interact with Pds5, which like Mis4 is a predicted suprahelical repeat protein (Figure 7B). We then tested whether the cohesin loader can bridge an interaction between the hinge and Psc3. However, we found that Psc3 by itself interacted with the cohesin hinge (Figure 7C). Addition of the cohesin loader resulted in the formation of a supramolecular complex containing Psc3, the cohesin loader, and the cohesin hinge. These results show that the cohesin hinge engages with Psc3, which lies in vicinity of the SMC heads. With its dual interactions, the cohesin loader likely facilitates this engagement. An interaction of the human Psc3 ortholog, SA1, with the cohesin hinge has also recently been detected (Huis in ’t Veld et al., 2014). The resulting proximity of the SMC heads and hinge will twist a planar cohesin ring into a folded conformation. This is prone to expose the DNA sensor on the Psm3 head, which otherwise points into the inside of the cohesin ring and which might be crucial for the DNA-entry reaction. In addition, the Psm1-Psm3 hinge shows affinity to DNA, which could align it with the DNA sensor (Figures 7D and S6A). The implications for cohesin loading are discussed below.

## Discussion

### A Unified Model for DNA Entry into and Exit out of the Cohesin Ring

Our present study has advanced the biochemical characterization of the fission-yeast cohesin complex. Inclusion of the Pds5 and Wapl subunits, in addition to the cohesin tetramer core, has allowed us to gain a fuller picture of topological cohesin loading onto DNA as well as its subsequent unloading. Together, these reactions recapitulate the dynamic DNA association of the cohesin ring that is characteristic for the behavior of the complex in vivo. Our analysis revealed surprising parallels between DNA transit in both directions.

Let us first consider the DNA exit reaction, which appears well mapped out by what we now know. DNA inside the cohesin ring is ideally placed to make contact with the SMC heads, akin to what is seen in the crystal structure of DNA bound to the Rad50 SMC heads (Rojowska et al., 2014). This engages the DNA sensor, consisting of two conserved lysines on the Psm3 head, which in turn triggers ATP hydrolysis. We envision that DNA contact is conveyed to the ATPase via an arginine finger that emanates from a peptide loop directly underneath the DNA sensor and that reaches down to contact ATP (Lammens et al., 2004, Lengronne et al., 2006). ATP hydrolysis weakens the SMC head interaction, thereby opening an outward path for the DNA (Figure 7D, “unloading”). Passage through the SMC heads is the first of two steps required for DNA to become free. The kleisin doubles up the SMC head interaction, thus Wapl-facilitated dissociation of the Rad21 N terminus from Psm3 completes the DNA exit reaction. The Rad21 N terminus is displaced only once ATP is bound and therefore most likely the SMC head interface has closed again. The DNA thus passes two interlocking gates, only one of which can be open at any time.

How does DNA exit relate to the DNA-entry reaction into the cohesin ring? The DNA-sensing lysines, ATP hydrolysis, as well as Wapl all contribute to entry as they do to exit. This opens the possibility that DNA transit follows the same trajectory, both into and out of the cohesin ring. A prerequisite for this is that DNA can engage the DNA sensor from the outside; however, in the planar ring configuration, the two lysines point inward. This is where the cohesin loader comes into play, making contacts both at the SMC hinge as well as close to the SMC heads. These contacts, together with an interaction that we have detected between the hinge and Psc3, are prone to induce a conformational change that exposes the DNA sensor. One could think of it as folding the cohesin ring “inside-out” (Figure 7D, “loading”). We note that the proposed cohesin folding is consistent with the bent conformation seen during atomic force microscopic observations of the Psm1-Psm3 dimer (Sakai et al., 2003). A discernible FRET signal between fluorophores at the budding-yeast cohesin hinge and the Pds5 subunit, next to the SMC heads (Mc Intyre et al., 2007), suggests that such a folded state indeed occurs in vivo. Once DNA contacts the DNA sensor from the outside, the same sequence of events, ATP hydrolysis-dependent passage through the heads, then Wapl-facilitated passage past the Rad21N-Psm3 interface, will lead to DNA entry into the cohesin ring. DNA transit through the ring perimeter using the same trajectory thus alternatingly leads to DNA entry or exit.

Although DNA entry and exit consist of the same reactions, the ATPase requirements to achieve them are distinct. This is likely due to the differing geometries of cohesin during DNA entry and exit. Head disengagement, although essential, is not the limiting step during loading. Rather, the substantial conformational change of cohesin in preparation for loading is likely to be rate limiting. That conformational change might include a tendency to disrupt the SMC head interaction, such that ATP hydrolysis becomes possible even when the ATPase is compromised, e.g., by Walker B mutations or if calcium replaces magnesium. On the contrary, once DNA is inside the ring, its direct access to the lysine sensors means that DNA exit becomes limited by cohesin’s DNA-dependent ATPase. This can explain the greater sensitivity of the unloading reaction to alterations in the ATPase. (Our finding that ATP hydrolysis during unloading can be replaced by prolonged incubation without nucleotide reflects an alternative, probably unphysiological, way to achieve head disengagement.) Differences between the ATPase requirements for DNA entry and exit might be instrumental when it comes to regulating the relative rates of both reactions.

### Alternative Models for Cohesin Loading

Our model for DNA entry into and exit out of the cohesin ring by a common DNA trajectory is consistent with many previous observations. The model naturally explains why the absence of Wapl both leads to lower levels of cohesin on chromosomes and at the same time stabilizes cohesin on DNA (Bernard et al., 2008, Lopez-Serra et al., 2013, Rowland et al., 2009, Sutani et al., 2009). Wapl is not essential for cohesin loading, suggesting that following passage through the SMC heads, DNA becomes topologically trapped in a gap between the SMC heads and the kleisin. In the absence of Wapl, occasional spontaneous opening of the Rad21N-Psm3, or Rad21C-Psm1, interface could complete loading at a lower rate. In budding yeast, stable Smc1-kleisin interaction requires ATP, suggesting that this interaction might sometimes loosen (Arumugam et al., 2003, Weitzer et al., 2003). These considerations explain why a covalent fusion of the Psm3 head with the kleisin N terminus compromises, but does not abolish, cohesin function. Although such a fusion will be unable to block loading, it will substantially hinder cohesin unloading from DNA, consistent with the available observations (Buheitel and Stemmann, 2013, Gruber et al., 2006).

Wapl facilitates cohesin loading even in the absence of Mis4-Ssl3. The dependence on the DNA-sensing lysines suggests that Wapl-facilitated loading also starts with DNA contact at the SMC heads. A loading-competent cohesin conformation might be adopted less frequently in the absence of the cohesin loader, but accelerated passage past the Rad21N-Psm3 gate, or an additional function of Wapl, might compensate for the disadvantage. We cannot exclude an alternative explanation for Wapl-facilitated cohesin loading, involving DNA entry following a reverse trajectory, i.e., passing the Rad21N-Psm3 gate first and then the heads (Figure S6B, “Rad21N-Psm3 entry”). This notion is consistent with efficient DNA loading by Pds5 and Wapl in the presence of non-hydrolyzable ATP, as the first loading step would be expected to depend on the presence of ATP but not its hydrolysis. If this is the case, then the conserved Psm3 lysines must play a role in facilitating Pds5- and Wapl-dependent Rad21N-Psm3 disengagement, in addition to being a DNA sensor that controls ATP hydrolysis.

It has been suggested that loading of cohesin onto chromosomes involves opening of its SMC hinge (Buheitel and Stemmann, 2013, Gruber et al., 2006, Nasmyth, 2011). This proposition is based on the observation that ligand-induced dimerization of domain insertions at the SMC-hinge interferes with cohesin loading. Another plausible interpretation of this result is that the hinge insertions interfere with protein-protein or protein-DNA interactions that are part of the DNA-entry reaction. Nevertheless, we cannot formally exclude the possibility that ATP hydrolysis conveys a conformational change to the SMC hinge that leads to its opening (Figure S6B, “hinge entry”). How the same ATPase in this case fuels fundamentally different reactions during DNA entry and exit would need explanation. Of note, if ATP hydrolysis indeed led to hinge opening for DNA entry, head dissociation would have to be actively prevented, or else DNA would merely pass through both hinge and heads but not become entrapped. In an attempt to settle whether hinge opening occurs during cohesin loading, we engineered cysteine-directed chemical hinge crosslinks. However, the chemical crosslinkers interfered with in vitro loading even of unmodified fission-yeast cohesin. Future experiments with a cysteine-free complex should be able to settle whether or not hinge opening is part of the cohesin-loading reaction.

### Implications for Cohesion Establishment

Our results offer an explanation of how acetylation during cohesion establishment stabilizes cohesin on DNA (Rolef Ben-Shahar et al., 2008, Chan et al., 2012, Lopez-Serra et al., 2013, Ünal et al., 2008). Lysine acetylation, in analogy with replacing lysines by glutamines in our experiment, can be expected to prevent DNA from stimulating ATP hydrolysis. This will block the first step during both the cohesin-loading and -unloading reactions. It is a commonly held view that cohesin acetylation counteracts Wapl. Our results suggest that cohesin acetylation in fact blocks a very early step in DNA entry and exit such that Wapl is deprived of a substrate that it could act on. This explains how acetylation stabilizes cohesin in a robuster way, compared to what is achieved by removing Wapl (Guacci et al., 2015, Lopez-Serra et al., 2013). The realization that acetylation, like our lysine-to-glutamine changes, likely blocks both the DNA-entry and -exit reactions emphasizes the importance of accurate spatial and temporal regulation of cohesin acetylation. To establish enduring sister-chromatid cohesion, acetylation must occur soon after replication-fork passage but after all necessary DNA-binding reactions required to entrap sister chromatids are complete.

### Implications for the SMC Complex Family

Our results with fission-yeast cohesin might well be applicable to most SMC complexes that could load onto and unload from DNA in a similar way. A folded conformation of fission-yeast condensin has also been seen (Yoshimura et al., 2002), and a contribution of the SMC hinge to ATP hydrolysis and DNA binding is known in the case of bacterial condensin (Hirano and Hirano, 2006, Uhlmann and Hopfner, 2006). A head-hinge interaction of the Smc5-Smc6 complex, in turn, could be mediated by its suprahelical repeat subunits Nse5-Nse6, which have been variably reported to associate with both the SMC head and hinge (Duan et al., 2009, Palecek et al., 2006). In contrast to most SMC complexes, a dedicated loader is only known for cohesin. Even cohesin can load onto DNA independently of a loader in vitro, albeit with low efficiency. What singles out cohesin might be its relatively stable mode of DNA binding. Because loading and unloading are linked, a slow off-rate means also a slow on-rate, which could set a requirement for a cohesin loader. The cohesin loader’s primordial function might be related to nucleosome remodeling (Lopez-Serra et al., 2014), whereas the physical interactions with cohesin are an acquired adaptation.

Given the conservation of kleisins, the mechanism by which DNA passes two sequential gates to enter or exit is likely conserved among SMC complexes. Such an interlocking mechanism could facilitate sequential rounds of DNA entry. It would allow cohesin and other SMC complexes to load onto a second DNA strand, without the risk of losing association with the first. Although we have expanded our understanding of how DNA enters into and exits from the cohesin ring, how a second strand is captured is a key question for future studies.

## Experimental Procedures

### Proteins and DNA

Fission-yeast Pds5 was expressed as an E2a epitope, PreScission protease recognition sequence, and Protein A fusion protein in budding yeast and purified by sequential column chromatography on IgG-agarose, Capto Q, and gel filtration (GE Healthcare). Recombinant Wapl was expressed as GST, PreScission protease recognition sequence, and E2a epitope fusion protein and purified from *E. coli* by glutathione sepharose and heparin chromatography. Protein A and GST tags were removed by protease cleavage during purification steps. All cohesin derivatives, Psc3, Mis4-Ssl3, and DNA substrates for the in vitro assays were prepared and purified as described (Murayama and Uhlmann, 2014). Details can be found in the Supplemental Experimental Procedures.

### Biochemical Assays

In vitro cohesion-loading assays were carried out essentially as described (Murayama and Uhlmann, 2014). If not stated otherwise, cohesin tetramers (150 nM), supplemental Psc3 (100 nM), and Mis4-Ssl3 (100 nM) were mixed with 3.3 nM relaxed circular DNA (RC-DNA) in CL buffer (35 mM Tris-HCl [pH 7.5], 1 mM tris [2-carboxyehyl] phosphine [TCEP], 30 mM NaCl, 1 mM MgCl_2_, 15% [v/v] glycerol, and 0.003% Tween 20). Reactions were initiated by addition of 0.5 mM ATP and incubated at 32°C. Cohesin-DNA complexes were recovered by immunoprecipitation, and cohesin-bound DNA was analyzed by agarose-gel electrophoresis.

For cohesin-unloading reactions, a sequential unloading incubation was initiated by addition of an equal volume of Pds5 (100 nM) and Wapl (100 nM), which were preincubated at 32°C for 5 min in CL buffer containing 270 mM NaCl, so that the final NaCl concentration in the unloading reaction became 150 mM. The reactions were further incubated at 32°C, and cohesin-bound DNA was analyzed as described above. Alternatively, cohesin was immunopurified following the loading reaction, and bead-bound cohesin-DNA complexes were incubated with Pds5 and Wapl. Details of these reactions and other procedures, including ATPase, immunoprecipitation, and DNA-binding assays are described in the Supplemental Experimental Procedures.

## Supplemental Information

Document S1. Supplemental Experimental Procedures

Document S2. Article plus Supplemental Information
