# Supplementary material for: DNA Entry into and Exit out of the Cohesin Ring by an Interlocking Gate Mechanism
Source: Cell. 2015 Dec 17;163(7):1628–40. doi: 10.1016/j.cell.2015.11.030 (PMC4701713; doi:10.1016/j.cell.2015.11.030)
Supplement: Document S1. Supplemental Experimental Procedures [file mmc1.pdf]

Cell

Supplemental Information

DNA Entry into and Exit out of the Cohesin Ring by an Interlocking Gate Mechanism

Yasuto Murayama and Frank Uhlmann

SUPPLEMENTAL EXPERIMENTAL PROCEDURES

Purification of Pds5

The *Schizosaccharomyces pombe pds5⁺* complementary DNA (cDNA) was amplified by PCR from a meiotic cDNA library (Yeast National BioResource Project, Osaka, Japan). The *pds5⁺* cDNA sequence was fused with an E2a epitope tag, PreScission protease recognition sequence, followed by a protein A tag and cloned into the budding yeast shuttle vector YIplac204 (*TRP1*) under the control of *GAL1* promoter. The plasmid YIp-*pds5* was cut with EcoRV and integrated into the budding yeast (*MAT***a**, *ade2-1, trp1-1, can1-100, leu2,3,112, his3-11,15, ura3-52, pep4Δ::HIS3MX*). The resultant strain was cultivated in YP broth containing 2% raffinose at 30°C until the optical density at 600 nm reached 2.0. Pds5 protein expression was induced by addition of galactose to a final concentration of 2% and the cells were grown for 4.5 h. The cells were harvested, washed and resuspended in an equal volume of CLH buffer (50 mM HEPES-KOH (pH 7.5), 1 mM dithiothreitol (DTT), 20% (v/v) glycerol, 300 mM NaCl, 0.5 mM phenylmethylsulfonyl fluoride (PMSF) and protease inhibitor cocktail (Roche)) at 4°C. The suspension was dropped into liquid nitrogen. Cells were disrupted by grinding the frozen cell pellets using a freezer mill (SPEX CertiPrep 6850). The resulting cell powder was thawed on ice and twice the cell volume of CLH buffer was added. The cell lysate was clarified by centrifugation at 45,000 x g for 30 min, followed by 200,000 x g for 1 h. 1 ml resin slurry per 50 ml lysate of IgG-agarose (Sigma) was added and mixed for 3 hours, then packed into a column (Bio-Rad). The IgG-agarose was washed with 10 bed volumes of CLH buffer, followed by 20 bed volume of H buffer (25 mM HEPES-KOH (pH 7.5), 0.5 mM TCEP and 10% glycerol) containing 300 mM NaCl. The resins were resuspended in 5 volume of H buffer containing 300 mM NaCl, 10 μg/ml RNaseA and 5 U/ml of PreScission protease (GE healthcare) and incubated at 4°C overnight (> 12h). The eluate was pooled and diluted with R buffer (20 mM Tris-HCl (pH 7.5), 0.5 mM TCEP and 10% glycerol) to a final NaCl concentration of 100 mM. The diluted eluate was applied to a Capto Q resin (1 ml, GE healthcare) and bound proteins were eluted in R buffer with a linear gradient from 100 mM to 1 M NaCl. The peak fractions were pooled and loaded onto a gel filtration column (Superdex 200 Increase, GE healthcare). The column was developed with R buffer containing 200 mM NaCl and the peak fractions were pooled and concentrated by ultrafiltration (Amicon Ultra, Millipore). Aliquots were flash frozen in liquid nitrogen and stored at -80°C. Typically, 500 μg of Pds5 was obtained from 50 g of budding yeast cells.

Purification of Wapl

The *wpl1*$^{+}$ cDNA was also PCR amplified from the meiotic cDNA library. The *wpl1*$^{+}$ gene was fused to an E2a tag at the N terminus and cloned into the GST fusion protein expression vector pGEX6P-1 (GE Healthcare). The plasmid pGEX-*wapl* was introduced to *E. coli* BL21-CodonPlus (DE3) (Stratagene). Fresh transformants were cultivated in Luria-Bertani (LB) medium containing 100 μg/ml ampicillin and 34 μg/ml chloramphenicol at 37°C until the optical density at 600 nm reached 0.5. Isopropyl β-D-1-thiogalactopyranoside (IPTG) was added to the culture to a final concentration of 0.5 mM and further grown at 18°C over night (approximately 15 hours). The cells were collected at 4°C, resuspended in H buffer containing 300 mM NaCl and protease inhibitor cocktail and disrupted by sonication. The cell lysate was clarified by centrifugation at 45,000 x g for 1 hour. The clarified lysate was mixed with glutathione sepharose (1 ml resin slurry, GE healthcare) for 3 h and packed into empty open column. The resin was washed with 30-bed volumes of H buffer containing 300 mM NaCl. The bound protein was eluted by addition of 5-bed volume of the same buffer containing 20 mM glutathione. PreScission protease was added to the eluate to a final concentration of 5 U/ml and incubated at 4°C for overnight (>12 h). The eluate was applied to a HiTrap Heparin HP column (GE healthcare) and bound proteins were eluted in R buffer with a linear gradient from 100 mM to 1 M NaCl. The peak fractions were pooled and mixed with 1/40 volume of glutathione sepharose for 20 min to remove residual GST proteins. The unbound fractions were pooled and concentrated by ultrafiltration. Aliquots were frozen in liquid nitrogen and stored at -80°C. Typically, 250 μg of Wapl protein was obtained from 3 liters culture.

Purification of the Psm1-Psm3 hinge

The cDNA sequences of the Psm1 (aa451 - 738) and Psm3 hinge (aa484 - 686) were amplified and cloned into pET28a and pET21b, respectively. To the Psm1 hinge, a 7 x histidine tag was fused at the C-terminus. The resultant plasmids were co-introduced into *E. coli* BL21-CodonPlus (DE3). Fresh transformants were grown in LB medium containing 100 μg/ml ampicillin, 50 μg/ml kanamycin and 34 μg/ml chloramphenicol at 37°C to an optical density at 600 nm of 0.5. IPTG was added to the culture to a final concentration of 0.5 mM and further grown at 18°C over night (approximately 15 hours). The cells were collected at 4°C, resuspended in H buffer containing 300 mM NaCl and protease inhibitor cocktail and disrupted by sonication. The cell lysate was clarified by centrifugation at 45,000 x g for 1 hour. The clarified lysate was mixed with 1 ml Ni-NTA resin slurry (Qiagene) for 3 hours and packed into a column. The resin was washed with 30 bed volumes of H buffer containing 300 mM NaCl and 10 mM imidazole. The bound proteins were eluted by addition of 5 bed volumes of H buffer containing 300 mM NaCl and

400 mM imidazole. The eluate was loaded onto a Superdex 200 Increase column and developed with R buffer containing 300 mM NaCl. The peak fractions were collected, frozen in liquid nitrogen and stored at -80°C. Typically, 500 μg of the Psm1-Psm3 hinge dimer was obtained from 3 liters culture.

Purification of variant cohesin tetramers

A *psm1* Walker B mutant (E1161Q, denoted "1B"), *psm3* Walker B mutant (E1128Q, denoted "3B") and *psm3* acetylation site mutant cDNAs (K105Q and K106Q) were generated by site-directed mutagenesis. TEV protease cleavable *psm3-TEV* was created by inserting a Tobacco Etch Virus (TEV) protease recognition sequence (ENLYFQG) following D256 and Y945, each, in Psm3. The resultant *psm1* and *psm3* cDNAs were cloned into the budding yeast integrative vector YIplac211 (*URA3*) under control of the bidirectional *GAL1/10* promoter in the *GAL10* or *GAL1* direction, respectively. The various Psm1-Psm3 expression constructs, followed by a Rad21-Psc3 expression construct, were integrated into budding yeast for expression of the mutant cohesin complexes. These were expressed and purified by sequential purification steps on IgG-sepharose, heparin and gel filtration as described, as was the cohesin tetramer containing TEV protease cleavable Rad21 (Murayama and Uhlmann, 2014).

DNA and antibodies

Covalently closed circular DNA (cccDNA), relaxed circular DNA (RC-DNA) and linear DNA of pBluescript KSII (+) were prepared as described (Murayama and Uhlmann, 2014). The antibodies used for immunoprecipitation were anti-Pk (V5, AbD Serotec), anti-HA (12C5A, Roche), anti-hexameric histidine (Novogen), anti-E2a (5E11, Abcam) and anti-Rad21 (BioAcademia).

***In vitro* cohesin loading assay**

Standard reactions (15 μl) were carried out essentially as described (Murayama and Uhlmann, 2014). Given concentrations of the proteins, DNA and compounds denote final concentrations. Mis4-Ssl3 (100 nM), cohesin tetramer (150 nM), supplemental Psc3 (100 nM) and RC-DNA (3.3 nM molecules) were mixed in CL buffer (35 mM Tris-HCl (pH 7.5), 1 mM TCEP, 30 mM NaCl, 1 mM $MgCl_2$, 15% (v/v) glycerol and 0.003% Tween 20). The reactions were initiated by the addition of 0.5 mM ATP and incubated at 32°C for 60 min if not otherwise indicated. Pds5 and Wapl were used at 100 nM concentration and reactions including these proteins were carried on for 15 min. To terminate the reactions, 500 μl of CP buffer (35 mM Tris-HCl (pH 7.5), 1 mM TCEP, 500 mM NaCl, 10 mM EDTA, 5% (v/v) glycerol and 0.35% Triton X-100) was added and incubated for 10 min on ice. Anti-Pk IgG, prebound to Protein A conjugated

magnetic beads (Dynal) were added to the mixture and rocked at 4°C over night (15 hours). The beads were washed three times with CW1 buffer (35 mM Tris-HCl (pH 7.5), 1 mM TCEP, 750 mM NaCl, 10 mM EDTA and 0.35% Triton X-100) and once with CW2 buffer (35 mM Tris-HCl (pH 7.5), 1 mM TCEP, 100 mM NaCl, 10 mM EDTA and 0.1% Triton X-100). The cohesin-bound DNA was eluted by incubating the beads at 50°C for 20 min in 15 μl of elution buffer (10 mM Tris-HCl (pH 7.5), 1 mM EDTA, 50 mM NaCl, 0.75% SDS and 1 mg/ml protease K). The recovered DNA was analyzed by 1% agarose gel electrophoresis in TAE buffer. The gel was stained with GelRed (Biotum) and gel images were captured using a GelDoc-It Imager (UVP) and band intensities were quantified using ImageQuant.

***In vitro* cohesin unloading assay**

Cohesin loading reactions were performed as described above. Meanwhile, 200 nM of Pds5 and Wapl were incubated in 15 μl of CL buffer containing 270 mM NaCl and 0.5 mM ATP at 32°C for 5 min. The unloading reactions were initiated by combining 15 μl Pds5 and Wapl with 15 μl of the cohesin loading reaction and further incubation at 32°C for the indicated time. Note that the final concentrations were now 50 nM Mis4-Ssl3, 75 nM cohesin tetramer, 50 nM supplemental Psc3, 100 nM Pds5, 100 nM Wapl and 150 mM NaCl. The reactions were terminated and cohesin-bound DNA was analyzed as described above. When loading reactions were initiated using Pds5 and Wapl (100 nM each) instead of Mis4-Ssl3, then 100 nM of Pds5 and Wapl were used in the preincubation mix for the unloading reaction.

DNA release assay

RC-DNA bound cohesin was retrieved by anti-Pk immunoprecipitation from cohesin loading reactions as described above, but using CPR buffer (35 mM Tris-HCl (pH 7.5), 1 mM TCEP, 500 mM NaCl, 5% (v/v) glycerol and 0.2% Tween 20). The beads were washed twice with CWR buffer (35 mM Tris-HCl (pH 7.5), 1 mM TCEP, 750 mM NaCl and 0.2% Tween 20) and twice with PW buffer (35 mM Tris-HCl (pH 7.5), 1 mM TCEP, 150 mM NaCl, 1 mM $MgCl_2$, 15% (v/v) glycerol and 0.0025% Tween 20). The beads were resuspended in 15 μl of PW buffer containing 400 nM Pds5, 400 nM Wapl and 100 nM Psc3. The reaction was initiated by addition of 0.5 mM ATP and incubated at 32°C for 60 min. Supernatant (the released fraction) and beads (the cohesin-bound fraction) were deproteinized and analyzed as described above.

Confirmation of topological DNA entrapment by cohesin

Cohesin-bound cccDNA was captured and immobilized on magnetic beads as above. The beads were washed as described, but using CP and CW buffer lacking EDTA. The beads were washed once with RE buffer (35 mM Tris-HCl (pH 7.5), 0.5 mM TCEP, 100 mM NaCl, 10 mM $MgCl_2$, 0.1 mg/ml BSA and 0.1% Triton X-100). The beads were now incubated with PstI (20 U, NEB) in 10 µl of RE buffer and incubated at 32°C for 30 min with repeated tapping. The salt concentration was adjusted to 500 mM and further incubated at 4°C for 10 min. Supernatant and bead fractions were deproteinized and analyzed as described above.

Alternatively, DNA loading reactions were carried out with wild type, Psm3-TEV and Rad21-TEV cohesin tetramers as described above, but using 1 mM $CaCl_2$ instead of $MgCl_2$ to reduce enzymatic unloading. Cohesin-bound RC-DNA was retrieved and immobilized on magnetic beads as described above in the TW buffer (35 mM Tris-HCl (pH 7.5), 0.5 mM TCEP, 300 mM NaCl, 1 mM $CaCl_2$, 0.25 mM ATP and 0.1% Triton X-100). The beads were washed once with TW buffer, then once with TC buffer (35 mM Tris-HCl (pH 7.5), 0.5 mM TCEP, 100 mM NaCl, 1 mM $CaCl_2$, 0.25 mM ATP and 0.05% Tween 20). The resultant beads were treated with 5 units TEV protease in 10 µl of TC buffer at 16 °C for 1.5 h. The reactions were adjusted to 300 mM NaCl in 15 µl final volume in TC buffer and incubated on ice for 10 min. The supernatant (released fraction) and beads bound fraction (cohesin bound fraction) were deproteinized and analyzed by agarose gel electrophoresis as described above.

DNA electrophoretic mobility shift assay

The indicated concentrations of Pds5, Wapl, Psc3 or the Psm1-Psm3 hinge were incubated with 0.825 nM (molecules) PstI-linearized pBluescript KSII (+) at 32°C for 15 min in DB buffer (25 mM HEPES-KOH (pH7.5), 0.5 mM TCEP, 50 mM NaCl and 15% (v/v) glycerol). The reactions were analyzed by 0.8% agarose gel electrophoresis in TAE.

ATPase assay

Cohesin tetramer (150 nM), Pds5 and Wapl (100 nM, each) or Mis4-Ssl3 (100 nM) and RC-DNA (3.3 nM) were mixed in 15 µl of CL buffer containing the indicated concentration of NaCl. The reactions were initiated by addition of 0.25 mM ATP, spiked with [γ-^{32}P] ATP. Aliquots (1.5 µl) were taken at 0, 15, 30 and 60 min and reactions were terminated by addition of a 3-fold volume of 0.5 M EDTA. 1 µl of the samples were spotted on polyethylene imine cellulose F sheets (Merck) and developed by thin layer chromatography using 400 mM LiCl in 1 M formic acid as the mobile phase. The separated spots

representing ATP and hydrolyzed inorganic phosphate were quantified following phosphoimager analysis using ImageQuant.

Immunoprecipitation

The indicated concentrations of proteins were incubated in 100 µl IP buffer (25 mM Tris-HCl (pH 7.5), 0.5 mM TCEP, 100 mM NaCl, 0.5 mg/ml BSA, 5% glycerol and 0.05% NP-40). This included for the experiment shown in Figure 1C, 30 nM cohesin tetramer or trimer, 100 nM Pds5 and 100 nM Wapl, for Figures 7B and C, 100 nM Psc3-Pk, 100 nM Mis4-Ssl3 and 250 nM Smc hinge. The reaction mixtures were incubated at 30°C for 15 min then at 4°C for 1 hour. 5 µl of antibody-bound Protein A conjugated magnetic beads were added to the reactions and rocked at 4°C for 2 hours. The beads were washed twice with 500 µl of IP buffer and the precipitated proteins were analyzed by Western blotting.

In experiments that monitored dissociation of the Rad21N, cohesin containing TEV-cleavable Rad21 (50 nM) was incubated in 15 µl of CL buffer with Mis4-Ssl3 (100 nM) or Pds5 and Wapl (100 nM each) in the absence or presence of PstI-linearized DNA (1.1 nM) or indicated adenosine derivatives (0.5 mM) at 32°C for 5 min. AcTEV protease (2.5 U, Invitrogen) was added to the reaction and further incubated for 30 min. The reaction was terminated by addition of 500 µl of CP buffer and anti-Pk antibody, prebound to Protein A conjugated magnetic beads, was added and rocked to retrieve cohesin. The beads were then washed twice with CP buffer and once with CW2 buffer. The bound proteins were analyzed by Western blotting.
